# Insomnia Impairs Both the Pro-BDNF and the BDNF Levels Similarly to Older Adults with Cognitive Decline: An Exploratory Study

**DOI:** 10.3390/ijms24087387

**Published:** 2023-04-17

**Authors:** Sergio Sánchez-García, Karla Moreno-Tamayo, Ricardo Ramírez-Aldana, Carmen García-Peña, Raúl Hernán Medina-Campos, Paola García dela Torre, Nadia Alejandra Rivero-Segura

**Affiliations:** 1 Unidad de Investigación Epidemiológica y en Servicios de Salud, Área de Envejecimiento, Centro Médico Nacional Siglo XXI, Instituto Mexicano del Seguro Social, Mexico City 06720, Mexico; sergio.sanchezga@imss.gob.mx (S.S.-G.); kmoreno.gdl@gmail.com (K.M.-T.); 2 Dirección de Investigación, Instituto Nacional de Geriatría, Mexico City 10200, Mexico; ricardoramirezaldana@gmail.com (R.R.-A.); raulmdc81@gmail.com (R.H.M.-C.); 3 Dirección General, Instituto Nacional de Geriatría, Mexico City 10200, Mexico; mcgarcia@inger.gob.mx; 4 Unidad de Investigación Médica en Enfermedades Neurológicas, Centro Médico Nacional Siglo XXI, Instituto Mexicano del Seguro Social, Mexico City 06720, Mexico; pgarciatorre@gmail.com

**Keywords:** BDNF, insomnia, cognitive decline, aging, frailty

## Abstract

Sleep disorders, including insomnia, are common during aging, and these conditions have been associated with cognitive decline in older adults. Moreover, during the aging process, neurotransmitters, neurohormones, and neurotrophins decrease significantly, leading to the impairment of cognitive functions. In this sense, BDNF, the most abundant neurotrophic factor in the human brain, has been suggested as a potential target for the prevention and improvement of cognitive decline during aging; however, the current evidence demonstrates that the exogenous administration of BDNF does not improve cognitive function. Hence, in the present study, we quantified pro-BDNF (inactive) and BDNF (active) concentrations in serum samples derived from older individuals with insomnia and/or cognitive decline. We used linear regression to analyze whether clinical or sociodemographic variables impacted the levels of BNDF concentration. We observed that insomnia, rather than cognitive decline, is significantly associated with BDNF concentration, and these effects are independent of other variables. To our knowledge, this is the first study that points to the impact of insomnia on improving the levels of BDNF during aging and suggests that opportune treatment of insomnia may be more beneficial to prevent cognitive decline during aging.

## 1. Introduction

Neurotrophins are a family of small polypeptides (12–13 kDa) expressed in the brain and peripheral tissues that have been associated with the proliferation of neural progenitors, neuronal morphology, and synaptogenesis [1,2]. Particularly, the brain-derived neurotrophic factor (BDNF, mature peptide) is the most abundant neurotrophin in the human brain and is released as proBDNF (immature peptide, 32 kDa) for cleavage by membrane-associated or extracellular proteases [3]. Interestingly, both immature and the mature BDNF are neuroactive; for instance, proBDNF inhibits neurite growth, modulates synaptic plasticity via the induction or enhancement of long-term depression in the hippocampus, and induces apoptosis of specific neuronal cells during development and in adulthood by binding to p75TNR [4,5]. In contrast, BDNF promotes neuronal survival, cell migration, neurite growth, regulation of synaptogenesis, synaptic transmission, and neuronal plasticity and participates in learning and memory mechanisms (brain plasticity and cognitive function) via the tyrosine kinase receptor TrkB. However, the relative levels of proBDNF and mature BDNF are key regulators in modulating the structure and function of the central nervous system (CNS).

For example, impairments in BDNF concentration and expression are associated with neuropsychiatric diseases such as depression [6,7], as well as neurodegenerative diseases such as Alzheimer’s disease, Parkinson’s disease, and Huntington’s disease [8,9,10]. However, aging per se impairs BDNF levels [11], and aging has been identified as one of the main risk factors for the development of neurodegenerative diseases and dementias [12,13].

Additionally, alterations in plasmatic levels of BDNF are associated with mild cognitive impairment, an intermediate state between normal aging and early dementia [8]. Hence, BDNF has been suggested as a potential biomarker of impaired memory and general cognitive function in aging [14]. In fact, the neurotrophin hypothesis proposes that stress-related mental disorders result from a stress-induced decrease in BDNF expression [15]. In this sense, both primary and secondary sleep disorders have been associated with reduced serum BDNF levels [16,17] and with the subsequent development of cognitive impairment, as seen during aging [18,19].

Particularly, insomnia, defined as difficulty falling asleep or staying asleep [20,21], is the most prevalent sleep disorder in the adult population worldwide (more than 10%) [22]; and the prevalence increases in the aged population [23]. Moreover, insomnia is a risk factor for poor physical and mental health, increasing the likelihood to impair objective cognitive performance associated with the development of dementia [24], as well as triggering the onset and worsening of frailty during aging [25].

Nowadays, it is well known that insomnia influences, in part, BDNF levels. However, such reports are controversial since some indicate that individuals with acute sleep deprivation or major depression showed increased levels of BDNF, while individuals with chronic sleep deprivation display decreased BDNF levels [26,27,28]. Moreover, despite BDNF having been suggested as a potential indicator of insomnia [29], there are still missing epidemiological studies that aim to understand the directionality of the association between insomnia and BDNF, and other authors have mentioned that the literature on insomnia-related BDNF changes is limited [26]. Hence, in this study, we aim to address the missing information regarding the effect of insomnia on BDNF levels in serum samples of older adults, and then, we aim to understand the contribution of insomnia in the modulation of such neurotrophin by the association of the BDNF concentration with clinical and sociodemographic variables characterized in our cohort.

## 2. Results

As mentioned above, a total of 80 participants that met our selection criteria were included in this study. The sociodemographic characteristics and clinical variables of the participants are depicted in Table 1.

**Figure 1:**
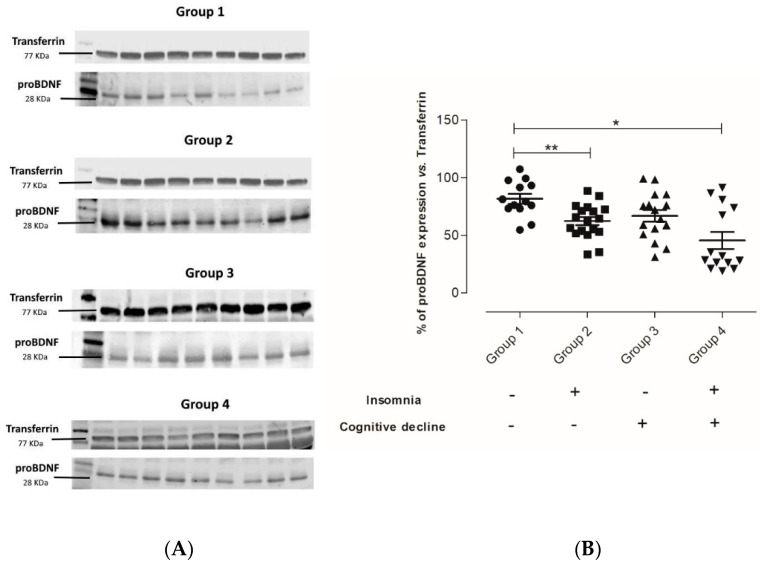
The effect of insomnia alone on the proBDNF content is comparable to the content observed in cognitive decline alone. Representative immunoblots for proBDNF content in serum samples according to the given classification (the uncropped immunoblots are available in the Supplementary Material Figure S1) (**A**). Densitometric analysis from the proBDNF immunoblots (**B**). n = 80; ** *p* = 0.0017 Group 1 vs. Group 2, * *p* = 0.0034 Group 1 vs. Group 4 One-ANOVA Post hoc test Dunns’ test. Circles correspond to Group 1 (Non-insomnia and non-cognitive decline, Control), squares correspond to Group 2 (Only insomnia), upward-pointing triangles correspond to Group 3 (Only cognitive decline), and downward-pointing triangles correspond to Group 4 (both insomnia and cognitive decline).

Once we classified the participants into the corresponding group, we aimed to determine the effect of insomnia and cognitive decline alone or combined on the proBDNF (the precursor of mature BDNF) content. The results from Figure 1 show that Group 2 had a significantly decreased proBDNF content in comparison to the control group (** *p* = 0.0017); however, the proBDNF estimated in Group 2 is comparable to Group 3, suggesting that insomnia alone may induce similar stress to cognitive decline alone on this precursor. Additionally, the proBDNF content in Group 4 is significantly decreased as compared to the control group (* *p* = 0.0034), and the trend is lower than in Groups 2 and 3, suggesting that the effect may be enhanced when both conditions are present.

**Figure 2:**
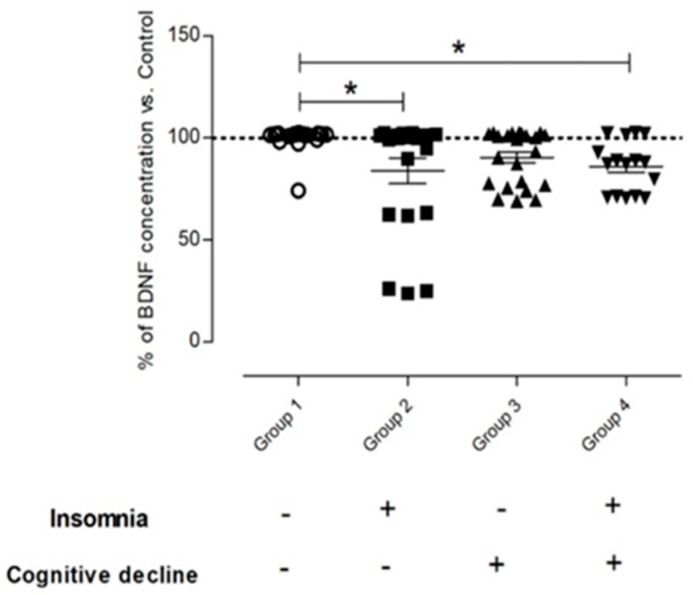
The BDNF concentration in individuals with insomnia is like those individuals with cognitive decline. This plot depicts the concentration of BDNF in serum samples of older individuals according to their group. Group 1 (n = 22) 9614.7 ± 571.5 pg/mL; Group 2 (n = 21) 8034.2 ± 2634.8 pg/mL; Group 3 (n = 23) 8691.3 ± 1257.9 pg/mL; Group 4 (n = 14) 8157.4 ± 1160.3 pg/mL. Group 1 vs. Group 2 * *p* = 0.0029; Group 1 vs. Group 4 * *p* = 0.0051. One-way ANOVA; post hoc test: Dunns’ test. Circles correspond to Group 1 (Non-insomnia and non-cognitive decline, Control), squares correspond to Group 2 (Only insomnia), upward-pointing triangles correspond to Group 3 (Only cognitive decline), and downward-pointing triangles correspond to Group 4 (both insomnia and cognitive decline).

Since the results demonstrate that the proBDNF content decreases in insomnia alone, cognitive decline alone, and in their combination, we aim to measure whether BDNF (the active form of the neurotrophin) concentration was also altered by insomnia. The results from the ELISA assay showed that individuals with insomnia alone (Group 2), or with the combination of insomnia and cognitive decline (Group 4) had significantly decreased BDNF concentrations as compared to controls (*p* = 0.0029; *p* = 0.0051, respectively). Moreover, the results also demonstrate that samples from Group 2 exhibit a similar concentration of BDNF as the samples from Group 3, corresponding to cognitive decline alone, suggesting that insomnia may be affecting BDNF synthesis and further processing in a similar way as cognitive decline does. Since the results from Figure 2 demonstrate that BDNF concentration decreases in both groups, insomnia alone or in cognitive decline alone, we perform a linear analysis to unveil the potential role of the clinical and sociodemographic variables on the BDNF levels.

From the linear analysis without controlling for any variables, we observed a negative significant association between insomnia alone (β = −1549.38, *p*-value = 0.004) and the co-existence of insomnia and cognitive decline (β = −1511.71, *p* = 0.011) with BDNF concentration, suggesting that people with insomnia alone or the combination of both (insomnia and cognitive decline) exhibit a decreased concentration of BDNF (Table 2).

After controlling for all variables in the model, including insomnia and cognitive decline, we observed that insomnia is negatively associated with BDNF concentration (β = −1899.98, *p* < 0.01), whereas cognitive decline was not significant. These results demonstrate that insomnia significantly decreases the concentration of this neurotrophin, suggesting that insomnia has a preponderant role in BDNF concentration rather than cognitive decline. Additionally, both pre-frail and frail individuals showed significant associations with BDNF (pre-frail, β = 1516.68, *p*-value = 0.024; frailty, β = 2218.53, *p*-value = 0.011) (Table 3). The model that included only insomnia and cognitive decline and the one including the covariates were significantly similar (LRT = 0.71, *p*-value = 1.0).

Moreover, when data were analyzed by groups, we observed a significant negative association between having insomnia alone (β = −2607.19, *p*-value = 0.001) and having both insomnia and cognitive decline (β = −2255.22, *p*-value = 0.008) compared to Group 1 (controls). We found a similar association between insomnia alone and Group 4 (the combination of insomnia and cognitive decline) and BDNF concentration. Moreover, frailty is another variable significantly associated with BDNF (pre-frail, β = 1514.293, *p*-value = 0.027; frailty, β = 2121.03, *p*-value = 0.017) (Table 4). The model including groups and the one including the covariates were significantly similar (LRT = 0.78, *p*-value = 1.0) (Table 4). Finally, since it is well known that anti-depressant intake increases BDNF levels, we also performed the association analysis excluding the participants that intake such medication. The results from these analyses were the same as the results depicted in both Table 3 and Table 4, suggesting that antidepressants did not significantly alter the BDNF levels in this study (Supplementary Material Table S1).

## 3. Discussion

As mentioned above, the neurotrophin hypothesis establishes that stress-related mental disorders result from a stress-induced decrease in BDNF expression [15]. Thus, the identification of such stressors and their appropriate management may be the keystone to preventing or delaying the appearance of mental disorders that lead to cognitive decline. In this context, it has been suggested that insomnia and other sleep disorders contribute to cognitive decline, neurodegenerative disease, and dementia [30]; however, the directionality of such an association and the molecular mechanisms that underlie these events is unclear. Hence, the current study focuses on firstly depicting the role of insomnia on BDNF concentration in older adults to increase the knowledge about insomnia-related BDNF changes (particularly in aging), and then, we aim to identify the contribution of insomnia as a stressor to decrease BDNF levels in older adults.

First, we found that BDNF concentration decreases significantly in samples derived from participants with insomnia alone (Group 2) and with the combination of insomnia and cognitive decline (Group 4). Surprisingly, when we analyzed the effect of cognitive decline alone (Group 3), we observed that the concentration of BDNF is slightly higher in comparison to Group 4. These results are in line with a previous study which demonstrated that insomnia decreases BDNF concentration [29]; however, the concentration that they report is double that we report in this study, and such a discrepancy may be attributed to the age of our participants, since, as mentioned above, aging per se decreases BDNF concentration.

On the other hand, since it is well established that the ratio of proBDNF and BNDF is crucial for the appropriate function of the CNS, here we addressed the effect of insomnia on proBDNF levels. In this sense, our data show that proBDNF levels also decrease significantly in both conditions, insomnia alone or in combination with cognitive decline; interestingly, this effect seems to be higher in the last condition. Such results are interesting since this is the first time that these conditions (insomnia and cognitive decline) have been reported to have an effect on proBDNF, leading to the suggestion that insomnia promotes changes in BDNF concentration from the synthesis of its precursor. Additionally, the literature mentioned that proBDNF and BDNF levels decrease in the pre-clinical stages of Alzheimer’s disease [31], and insomnia and cognitive decline are common risk factors associated with developing neurodegenerative diseases [32]; hence, our results may be considered for further studies to validate the potential use of proBDNF and BDNF levels as biomarkers for the classification of different stages of neurodegenerative diseases in older adults.

Additionally, our results from the linear analysis demonstrate that insomnia is associated with a decreased BDNF concentration in the serum of older adults, independent of the cognitive status, suggesting that sleep disturbances such as insomnia exert a significant influence on this neurotrophin. On the matter, it has been shown that exogenous administration of BDNF does not improve insomnia [33,34]; however, lifestyle alterations such as increased social interaction, mental stimulation, exercise, and a healthy diet enriched with polyphenols and flavonoids result in a longer-lasting recovery of BDNF levels and in better sleep quality [35,36]. In addition, the fact that insomnia significantly influences BDNF levels more than cognitive decline suggests that insomnia may be in part responsible for cognitive decline in the early stages; thus, insomnia may be considered as a potential therapeutic target for the prevention of cognitive decline in older adults.

On the other hand, although previous evidence has shown that serum BDNF is associated with cognitive decline [37,38], in our samples, we did not find a significant decrease in serum BDNF in older adults with cognitive decline alone. This discrepancy may be because we did not measure the severity of cognitive decline, and this is one of the main limitations of this study. Nevertheless, BDNF plasma levels have been reported to be significantly decreased in aged subjects, and a negative correlation between serum BDNF levels and age in healthy older adults has been reported [39,40]. Hence, serum BDNF association with cognition could be masked by aging in this population.

Overall, our results point to insomnia as a proper intervention to prevent cognitive decline by means of bringing BDNF to homeostasis in an aged population. Finally, it is essential to mention that despite the limitation of the small number of analyzed samples, this study contributes to understanding the role of insomnia in the cognitive state of elderly individuals and sheds light on insomnia to suggest this disorder as a potential target for the prevention and treatment of early stages of cognitive decline in aging. Additionally, since this is a cross-sectional study, our results only reflect the BDNF concentration in a single point, limiting our conclusions to the effect of insomnia in both BDNF concentration and proBDNF protein content, and further studies currently carried out by our research group are required to propose BDNF as a potential biomarker for the multiple aging outcomes.

## 4. Materials and Methods

### 4.1. Study Design and Participants

#### 4.1.1. Cohort Description

This is a cross-sectional study with information from the fifth wave (2018) of the “Cohort of Obesity, Sarcopenia, and Frailty of Older Mexican Adults” (COSFOMA). COSFOMA procedures and methods have previously been described in detail by Sánchez-García et al. [41]. Briefly, COSFOMA is a population-based prospective study that began in 2014 with the participation of 1252 adults aged 60 years or more who were beneficiaries of the Mexican Institute of Social Security (IMSS, by its Spanish acronym) in Mexico City. Participants were randomly selected from the IMSS administrative records. This cohort collected clinical and sociodemographic information annually through interviews carried out by trained health staff.

#### 4.1.2. Sample Collection

All the samples that were used in this study were collected between the months of April and March in 2018 at the Centro Médico Siglo XXI facilities. These samples were stored at −80 °C until their use. All the experiments and further analyses were conducted in August 2022. In this wave, only 523 (41.8%) participants agree to donate blood samples. However, for the aims of the present study, we only used samples 80 samples (6.4%) that were classified into four groups as follows:

Group (1) Non-insomnia and non-cognitive decline (control group)

Group (2) Only insomnia

Group (3) Only cognitive decline

Group (4) Both insomnia and cognitive decline

All cases found from the database were integrated and only for the control group, samples were randomly selected among those negative to both variables, insomnia and cognitive decline.

### 4.2. Insomnia Measure

The Athens Insomnia Scale (AIS) was used to measure insomnia symptoms through eight items scored on a four-point Likert scale. Total scores range from 0 to 24, with higher values indicating greater insomnia-related issues. The cut-off point of ≥6 points was used to indicate the presence of insomnia [40]. AIS has been validated in Mexico [40]. The AIS Cronbach alpha value in this study was 0.90.

### 4.3. Cognition Status Evaluation

Cognitive status was evaluated with the Mini-Mental State Examination (MMSE), a 30-item scale that examines different cognitive domains, with higher scores meaning better performance. A cut-off point adjusted by level of schooling (≤23) indicated cognitive decline [41,42]. It is important to note that the MMSE is a screening test used to support the clinical diagnosis of dementia, and the interpretation of the results should consider the context of the patient [43]. Here we used the MMSE as a tool for the identification of individuals with potential cognitive decline.

### 4.4. Medical and Sociodemographic Covariates

Sociodemographic variables included sex, age, years of education, and living arrangements. Health-related variables included current smoking, current alcohol consumption, multimorbidity, number of medications currently used, use of antidepressant medications, and frailty (Linda Fried score index).

Multimorbidity was defined as having two or more diseases out of a predefined list of conditions, namely: [44,45] hypertension, diabetes, heart disease, cancer, kidney failure, cerebrovascular disease, arthritis, chronic liver disease, and chronic pulmonary disease.

Medication intake. The use of antidepressant medications was considered separately because of the potential influence of these medications on modifying BDNF concentrations.

Anxiety symptoms were assessed with the Short Anxiety Screening Test (SAST), a scale developed to standardize the detection of anxiety in the elderly, including somatic symptoms, consisting of 10 items, scored from one to four points, with the highest possible score of 40 (highest anxiety level) [46].

Depressive symptoms were evaluated using the Center for Epidemiologic Studies Depression Scale (CES-D), a validated 20-item scale consisting of four factors: depressive effect, somatic complaints, positive impact, and interpersonal relations. Scores on the CES-D range from 0 to 60, where scores of 0 to 15 indicate the absence of depression and those of 16 to 60 reflect depressive symptomatology [47].

Frailty was assessed using the phenotype described by Fried et al. [39,48], briefly described as follows. Frail adults are defined as those who show three or more items from the following criteria: weight loss, self-reported exhaustion, low physical activity, slowness, weakness (low grip strength). Adults who show one or two criteria indicate a pre-frail condition, while the absence of these items means a non-frail state.

### 4.5. BDNF Immunoblots

Samples were prepared following the protocol previously reported in [49]. Briefly, serum samples were processed in RIPA buffer (cat# 20-188, Merck, Rahway, NJ, USA) supplemented with a 5 mM protease inhibitor cocktail (cat# 04693124001, Roche, Basel, Switzerland) and centrifuged at 2500 rpm for 30 min at 4 °C. The pellets were then discarded, and each sample’s aqueous phase was stored at −20 °C until their use. Protein concentrations were determined by BCA assay following the supplier’s instructions (cat# 23227, Thermo Fisher Scientific, Waltham, MA, USA). Then immunoblots were performed following the protocol reported in Rivero-Segura et al. [50]. Briefly, protein samples were re-suspended in loading buffer (cat#1610747, BioRad, Hercules, CA, USA) supplemented with 5% β-mercaptoethanol and denatured at 95 °C for 5 min in a block heater. Then, 40 μg of protein of each sample was loaded into 4–20% Mini-PROTEAN^®^ TGX Stain-Free™ Protein Gels (cat# 4568093, BioRad, Hercules, CA, USA) and run under denaturing conditions (90 mV for 1 h at RT°). Proteins were transferred to PVDF membrane (cat# GVWP04700, Merk, Rahway, NJ, USA) at 100 mV for 25 min in a cold bath, as previously reported in Rivero-Segura et al. [50]. Membranes were blocked with Intercept^®^ (PBS) Blocking Buffer (cat# 927-70001, LI-COR, Lincoln, NE, USA) for 1 H at RT°; then, membranes were incubated overnight at 4 °C with blocking buffer containing the corresponding primary antibody: anti-BDNF (cat# SC-546, Santa Cruz Biotechnology, Dallas, TX, USA) and anti-Trasnferrin (cat# ab82411, Abcam, Cambridge, UK), both kindly brought by Paola García dela Torre. The hybridized proteins were incubated with the corresponding secondary antibodies: anti-rabbit (cat # 926-32213 or cat#926-68073, LI-COR, Lincoln, NE, USA), conjugated with the fluorophore IRDye 800CW or IRDye^®^ 680RD. The signal was detected by fluorescence using the ODYSSEY blot-scanner (LI-COR). Densitometry analysis was performed with Image J software v. 2.9.0, and the protein content was normalized against transferrin.

### 4.6. BDNF ELISA

Mature BDNF (14 kDa) was quantified using the Human BDNF ELISA Kit (cat #. Ab212166, Abcam, Cambridge, UK) according to the supplier´s instructions. Briefly, samples were thawed once and diluted 1:10 in sterile water, each sample dilution was assayed in duplicate, and the plate was read at an endpoint at 450 nm. Results were calculated by interpolation into the standard curve and expressed as pg/mL.

### 4.7. Statistical Analysis

#### 4.7.1. For the Immunoblots and ELISA Assay

Data derived from both the immunoblots and the ELISA assay were analyzed with the GraphPad Prism^®^ software v 5. by one-way ANOVA followed by a Dunn´s post hoc test. Values are expressed as the mean ± SD. Significant differences were considered when *p* < 0.05.

#### 4.7.2. Association Analyses

Group was considered the explanatory variable, whereas the others were considered the control variables. BNDF was regarded as a response. A Kruskal–Wallis test was used to determine whether the response was similarly distributed between groups. Multiple comparisons were assessed through a Dunn’s test. The generalized linear models were fitted, considering BNDF as the response variable. A gamma distribution was considered since the response is skewed to the left. Identity and logarithmic link functions were used, obtaining similar results; thus, only the analysis using the former is presented. First, we fitted a model in which Group was used as an explanatory variable; after that, we created a model adding all the control variables, including, one at a time: frailty, balance, sarcopenia, and gait speed, which present collinearity between them. However, since similar results were obtained, only the analysis including frailty is presented. A similar model was fitted for comparison purposes, but using insomnia and cognitive decline instead of Group. A Likelihood Ratio Test (LRT) between the model, including only Group against the model, including all covariates already described, was performed. A similar test was performed when insomnia and cognitive decline were used instead of Group.

### 4.8. Ethical Statement

All participants and their legal guardians were informed of the research procedures and signed a letter of consent before participating. For illiterate participants, written informed consent was taken from their legal guardians. The COSFOMA protocol was approved by the IMSS National Committee Research (the National Committee for Scientific Research and the Ethics Committee on Health Research) (Registration No. 2012-785-067). Additionally, the use of samples for this study was approved by the Comité Nacional de Investigación Científica-IMSS with the number R-2017-785-107. All methods employed in the study were in accordance with the Declaration of Helsinki as well as guidelines from the Ley General de Salud of Mexico. The data are available upon express request addressed to the corresponding author and are currently in safekeeping by IMSS.

## 5. Conclusions

The present study is an exploratory analysis that points to the relevance of targeting insomnia in the health and the cognitive state of older adults, since, according to our results, insomnia impacts BDNF concentration independently from clinical or sociodemographic variables. Additionally, it is important to validate our results in independent cohorts to strengthen our findings.

## Figures and Tables

**Table 1:** Characteristics of the study population.

Clinical and Sociodemographic Variables	Data
Age, mean (SD), years	70.6 ± 6.3
Sex (%)	
Female (n, %)	57 (71.2)
Education (n, %)	12
None (Illiterate)	3 (3.75)
<6	11 (13.75)
From 6 to 9	22 (27.5)
From 10 to 11	12 (15)
12 and more	32 (40)
Living arrangement (accompanied) (n, %)	75 (93.75)
Multimorbidity (n, %)	20 (25)
Medication (SD) (n, %)	3.8 ± 2.9
Antidepressant consumption (n, %)	17 (21.3)
Anxiety (n, %)	17 (21.3)
Current smoking (n, %)	8 (10)
Current alcohol consumption (n, %)	14 (17.5)
Frailty	
Non-frail, n (%)	15 (18.8)
Pre-frail, n (%)	44 (55)
Frail, n (%)	21 (26.2)
Groups	
Group 1. (n, %)	22 (27.5)
Group 2. (n, %)	21 (26.3)
Group 3. (n, %)	23 (46.3)
Group 4. (n, %)	14 (17.5)
Total of participants (n, %)	80 (100)

SD = Standard Deviation, Group 1 (control) = Non-insomnia and non-cognitive decline, Group 2 = Only insomnia, Group 3 = Only cognitive decline, Group 4 = Both insomnia and cognitive decline.

**Table 2:** Unadjusted linear regression model for the association between insomnia and cognitive decline on BDNF concentration in serum.

BDNF (pg/mL)	β	S.E.	Z	*p*-Value	95% CI
Group 2	−1549.38	540.99	−2.86	0.004	−2609.71, −489.05
Group 3	−923.40	547.86	−1.69	0.092	−1997.20, 150.40
Group 4	−1511.72	597.09	−2.53	0.011	−2681.99, −341.44

Group 2 = Only insomnia, Group 3 = Only cognitive decline, Group 4 = Both insomnia and cognitive decline.

**Table 3:** Adjusted linear regression model for the association between variables separately considering insomnia and cognitive decline on BDNF concentration in serum.

BDNF (pg/mL)	β	S.E.	Z	*p*-Value	95% CI
Group 2	−1899.98	538.93	−3.53	0	−2956.3, −843.7
Group 3	−277.13	529.08	−0.52	0.6	−1314.10, 759.83
Anxiety	95.05	82.68	1.15	0.25	−67.01, 257.11
Depression	6.17	16.07	0.38	0.701	−25.33, 37.68
Sex (female)	−70.77	595.82	−0.12	0.905	−1238.6, 1097.02
Age	−15.34	39.50	−0.39	0.698	−92.77, 62.08
Education (years)					
<6	−847.65	1411.46	−0.6	0.548	−3614.08, 1918.77
From 6 to 9	−148.17	1276.71	−0.12	0.908	−2650.47, 2354.13
From 10 to 11	−95.63	1427.79	−0.07	0.947	−2894.06, 2702.79
12 and more	453.38	1346.30	0.34	0.736	−2185.32, 3092.09
Living arrangement (accompanied)	50.67	1027.22	0.05	0.961	−1962.65, 2063.99
Multimorbidity	−536.48	731.29	−0.73	0.463	−1969.796, 896.82
Number of Medications	121.30	92.988	1.3	0.192	−60.95, 303.55
Anti-depressants intake	−588.12	595.37	−0.99	0.323	1755.04, 578.8
Current smoking	267.24	858.20	0.31	0.755	−1414.79
Current alcohol consumption	892.10	671.18	1.33	0.184	−423.381
Frailty					
Pre-Frail	1516.68	673.65	2.25	0.024	196.34
Frail	2218.53	876.15	2.53	0.011	501.30

Group 2 = Only insomnia, Group 3 = Only cognitive decline.

**Table 4:** Adjusted linear regression model for the association between considered groups including insomnia and cognitive decline on BDNF concentration in serum.

BDNF (pg/mL)	β	S.E.	Z	*p*-Value	95% CI
Group 2	−2607.19	818.24	−3.19	0.001	−4210.90, −1003.47
Group 3	−913.85	762.66	−1.2	0.231	−2408.64, 580.94
Group 4	−2255.22	845.88	−2.67	0.008	−3913.11, −597.31
Anxiety	91.64	85.21	1.08	0.282	−75.35, 258.64
Depression	12.69	17.63	0.72	0.472	−21.90, 47.26
Sex (female)	−238.99	628.61	−0.38	0.704	−1471.06, 993.06
Age	−26.49	41.16	−0.64	0.52	−107.17, 54.2
Education (years)					
<6	−466.79	1481.46	−0.32	0.753	−3370.42, 2436.83
From 6 to 9	351.28	1380.56	0.25	0.799	−2354.57, 3057.15
From 10 to 11	243.63	1481.30	0.16	0.869	−2659.67, 3146.94
12 and more	809.96	1414.57	0.57	0.567	−1962.55, 3582.50
Living arrangement (accompanied)	13.76	1064.13	0.01	0.99	−2071.90, 2099.43
Multimorbidity	−602.12	740.20	−0.81	0.416	−2052.90, 848.65
Number of Medications	124.371	95.03	1.31	0.191	−61.88, 310.63
Anti-depressants intake	−529.49	613.03	−0.86	0.388	−1731.03, 672.03
Current smoking	111.17	890.10	0.12	0.901	−1633.40, 1855.74
Current alcohol consumption	602.13	736.52	0.82	0.414	−841.43, 2045.70
Frailty					
Pre-Frail	1514.29	683.61	2.22	0.027	174.43, 2854.15
Frail	2121.03	885.91	2.39	0.017	384.67, 3857.40

Group 2 = Only insomnia, Group 3 = Only cognitive decline, Group 4 = Both insomnia and cognitive decline.

## Data Availability

The data are available upon express request addressed to the corresponding author.

## Supplementary Materials

The supporting information can be downloaded at: https://www.mdpi.com/article/10.3390/ijms24087387/s1.
