# Supplementary material for: Insomnia Impairs Both the Pro-BDNF and the BDNF Levels Similarly to Older Adults with Cognitive Decline: An Exploratory Study
Source: Int J Mol Sci. 2023 Apr 17;24(8):7387. doi: 10.3390/ijms24087387 (PMC10139029; doi:10.3390/ijms24087387)
Supplement: Supplementary file 1 [file ijms-24-07387-s001.zip › ijms-2318471-supplementary.pdf]

Supplementary material Figure 1S: Uncropped immunoblots of pro-BDNF and Transferrin corresponding to the representative images showed in Figure 1 in the main manuscript. Figure 1S and 2S correspond to the samples of Group 1) No insomnia/no cognitive decline (control group); Figures 3S and 4S correspond to samples of Group 2 (Insomnia-alone); Figures 5S and 6S correspond to samples of Group 3 (Cognitive decline alone), and Figure 7S and 8S correspond to Group 4 (Insomnia and cognitive decline).

**Figure**	**Description**
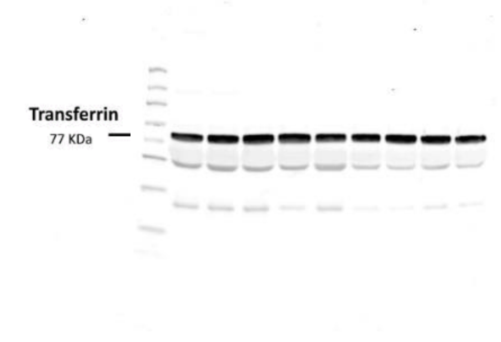	**Figure 1S.** Transferrin immunoblot representative of Group 1 (Control).
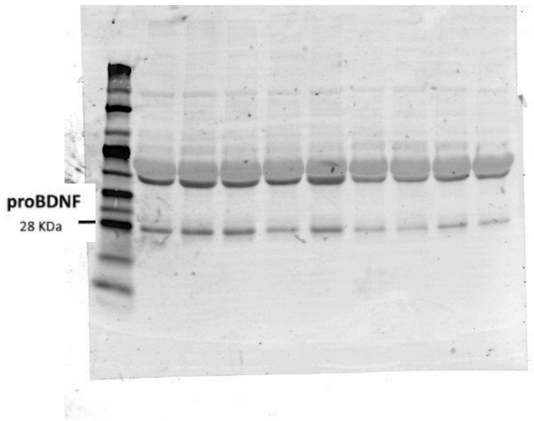	**Figure 2S.** Pro-BDNF immunoblot representative of Group 1.
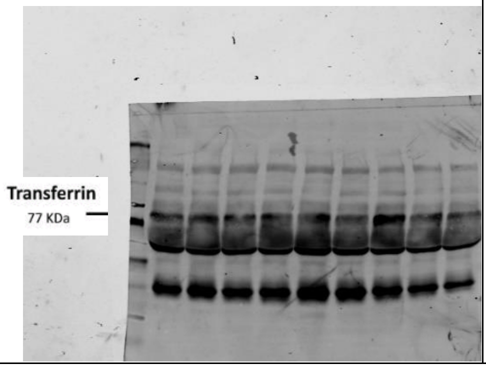	**Figure 3S.** Transferrin immunoblot representative Group 2 (Insomnia).

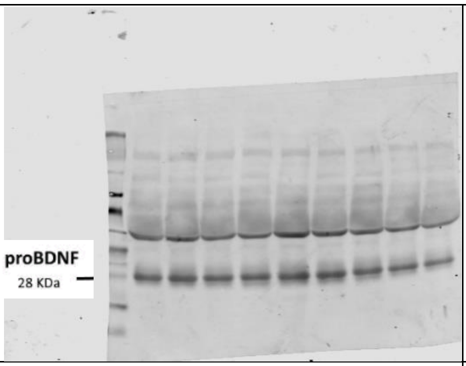

Figure 4S. Pro-BDNF immunoblot representative Group 2.

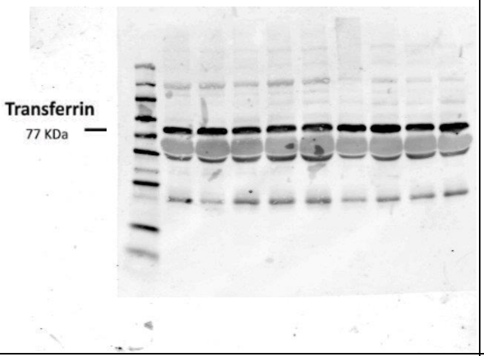

Figure 5S. Transferrin immunoblot representative of Group 3 (Cognitive decline).

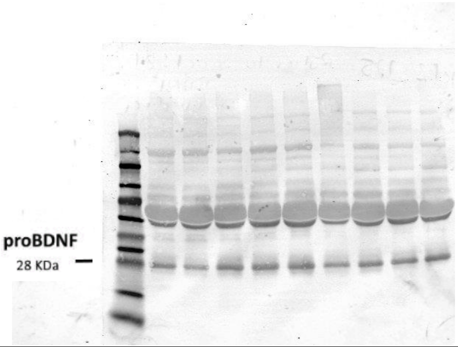

Figure 6S. Pro-BDNF immunoblot Representative of Group 3.

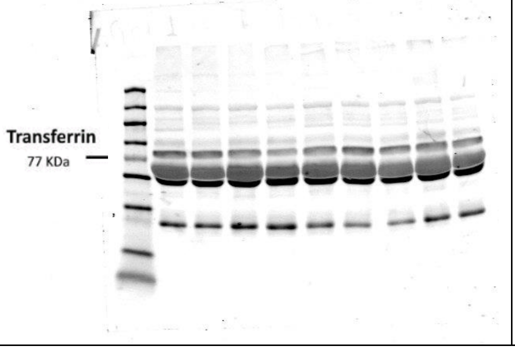

Figure 7S. Transferrin immunoblot representative of Group 4 (Insomnia and Cognitive decline).

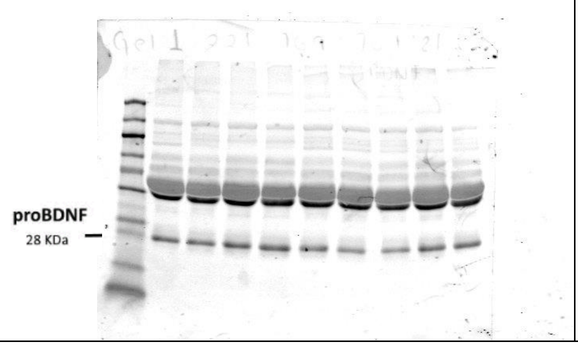

Figure 8S. Pro-BDNF immunoblot representative of Group 4.

Table 1S: Adjusted linear regression model for the association between variables separately considering insomnia and cognitive decline on BDNF concentration in serum excluding individuals that consume antidepressants.

BDNF (pg/mL)	β	S.E.	Z	p-value	95% CI
Group 2	-3925.26	1035.84	-3.79	0.000	-5955.48, -1895.05
Group 3	-1468.688	953.6808	-1.54	0.124	-3337.86, 400.49
Group 4	-2830.757	1125.707	-2.51	0.012	-5037.10, -624.41
Anxiety	123.2299	126.1338	0.98	0.329	-123.98, 370.44
Depression	32.77716	25.20955	1.30	0.194	16.63, 82.18
Sex (female)	-912.6268	809.3134	-1.13	0.259	-2498.85, 673.59
Age	-49.63	57.97761	-0.86	0.392	-163.26, 63.99
Education (years)					
<6	-1150.4	2503.48	-0.46	0.646	-6057.17, 3756.28
From 6 to 9	380.267	2442.991	0.16	0.876	-4407.90, 5168.44
From 10 to 11	-1041.0	2524.05	0.41	0.680	-5988.095, 3906.0
12 and more	186.22	2447.22	0.08	0.939	-4610.25, 4982.70
Living arrangement (accompanied)	6.86	1426.48	0.00	0.996	2788.984, 2802.71
Multimorbidity	-760.3275 -	996.7911	-0.76	0.446	2714.002, 1193.34
Number of Medications	161.0125 -	121.5344	1.32	0.185	77.19, 399.21
Anti-depressants intake			**ommited**		
Current smoking	-920.7924 -	1167.654	-0.79	0.430	3209.351, 367.76
Current alcohol consumption	469.0985 -	941.2218	0.50	0.618	1375.66, 2313.85
Frailty					
Pre-Frail	1570.69 -	840.4509	1.87	0.062	76.56317, 3217.94
Frail	2616.327	1121.223	2.33	0.020	418.7693, 4813.88

Group 1 (control)= non-insomnia and or non-cognitive decline, Group 2= Only in-somnia, Group 3= Only cognitive decline, Group 4= Both insomnia and cognitive decline.
